# Transcriptome Profiling of the Pineapple under Low Temperature to Facilitate Its Breeding for Cold Tolerance

**DOI:** 10.1371/journal.pone.0163315

**Published:** 2016-09-22

**Authors:** Chengjie Chen, Yafeng Zhang, Zhiqiang Xu, Aiping Luan, Qi Mao, Junting Feng, Tao Xie, Xue Gong, Xiaoshuang Wang, Hao Chen, Yehua He

**Affiliations:** College of Horticulture, South China Agricultural University, Guangzhou, 510642, Guangdong, P.R. China; Key Laboratory of Horticultural Plant Biology (MOE), CHINA

## Abstract

The pineapple (*Ananas comosus*) is cold sensitive. Most cultivars are injured during winter periods, especially in sub-tropical regions. There is a lack of molecular information on the pineapple’s response to cold stress. In this study, high-throughput transcriptome sequencing and gene expression analysis were performed on plantlets of a cold-tolerant genotype of the pineapple cultivar ‘Shenwan’ before and after cold treatment. A total of 1,186 candidate cold responsive genes were identified, and their credibility was confirmed by RT-qPCR. Gene set functional enrichment analysis indicated that genes related to cell wall properties, stomatal closure and ABA and ROS signal transduction play important roles in pineapple cold tolerance. In addition, a protein association network of CORs (cold responsive genes) was predicted, which could serve as an entry point to dissect the complex cold response network. Our study found a series of candidate genes and their association network, which will be helpful to cold stress response studies and pineapple breeding for cold tolerance.

## Introduction

Abiotic stress, including drought, extreme temperatures, flooding and chemical toxicity, is the principal cause of crop failure worldwide, reducing average yields for most major crops by more than 50%[1]. Several studies have shown that global crop production needs to double by 2050 to meet the projected demands for the rising population, which seems hard to achieve and deserves deep concern[2]. Cold stress, which occurs annually with winter, is among the most intimidating forms of abiotic stress. It adversely affects plant growth and development and thereby limits the distribution of crop species[3]. The response to cold stress differs among species[4] and genotypes[5]. At low temperatures, hardy plants or cold-tolerant genotypes grow properly, while others are severely injured or die. Most economically important crops are sensitive to low temperature[6]. Therefore, it is essential to reveal the molecular mechanisms of crop plants in response to cold stress, which is helpful for breeding cold-tolerant crop plants, and accordingly reducing production loss and expanding crop cultivation areas.

High-throughput sequencing technologies, such as second-generation sequencing platforms such as Illumina and third-generation sequencing platforms such as PacBio, allow genomic investigation at an unprecedented pace. NGS-based RNAseq paves the way for the transcriptomic analysis of almost all species. In the last decade, RNAseq has been successfully employed to reveal the complex cold-response mechanisms in many plant species, such as *Arabidopsis thaliana*[7], rice (*Oryza sativa*)[8–11], rapeseed (*Brassica napus*)[12], and sugar beet (*Beta vulgaris*)[13]. However, no research has yet applied RNAseq to study the cold-response mechanism of pineapple, and limited information in molecular level currently hinders its breeding for cold-tolerance.

Pineapple (*Ananas comosus*), a member of the family Bromeliaceae, is an economically important fruit that is widely cultivated in tropical and sub-tropical areas. Pineapple is cold sensitive, and almost all pineapple varieties are injured after exposure to 4°C for 24 hours. However, because its shortest production cycle is 14 months, the crop must undergo cold stress at least once in its life cycle, especially in subtropical regions. During winter, growers need to suspend sheets or plastic over the crop to provide sufficient warmth and protection, which increases cost, reduces profit and is not sufficiently effective. A cold-tolerant genotype of the pineapple cultivar ‘Shenwan’ has been selected, which was able to grow normally after exposure to 4°C for up to 7 days. It is a valuable resource for the breeding of cold-tolerant pineapple.

The recent report on the genome of pineapple cultivar ‘F153’ regarding its drought-tolerant characteristics described pineapple as a model for studying the evolution of CAM photosynthesis[14], and it has greatly promoted research on pineapple and other CAM plants. So far, most studies of plant cold stress at the molecular level have focused on C3 and C4 plants, while research on CAM plants has remained scarce. In this study, a genome-wide transcriptional analysis of cold stress response was performed on the cold-tolerant genotype of the pineapple cultivar ‘Shenwan’. This study will aid in understanding the cold stress response in pineapple at the molecular level, enriching the genomic information about cold-tolerant mechanisms for pineapple and other CAM plants.

## Results

### cDNA library construction and RNA sequencing

Eight libraries were generated using the mRNA from two sample groups: control (grown at 26°C) and cold (exposed to4°C for 24h), both containing three biological replicates (‘control_1’, ‘control_2’, ‘control_3a’, ‘cold_1’, ‘cold_2’, ‘cold_3a’)and one technical replicate (‘control_3b’, ‘cold_3b’). In addition, equal amounts of mRNA from eight samples were pooled, generating the library marked ‘pool’. These cDNA libraries were then subjected to Illumina deep sequencing. In total, 76,096,690 paired-end and 99,944,633 single-end clean reads were obtained from the pool, control and cold libraries (S1 Table and S1 Fig).

### Transcript assembly

All clean reads were *de novo* assembled into 89,712 transcripts using Trinity[15]. A total of 45,207 representative transcripts (unigenes) was obtained with a total length of 37,269,296 bp after redundancy removal using CD-hit[16]. The length of the unigenes ranged from 201–15,104 bp, with N50 of1, 470bp. There were 21,767 unigenes (48.14%) in the length range of 300–1,200bp, 13,714 unigenes (30.33%) with length<300 bp, and 9,726 unigenes (21.44%) with length >1,200 bp (S2 Fig). CDS prediction analysis was also performed using Transdecoder from the Trinity package. Approximately 17,471 (73.11% out of all predicted CDS) CDSs had lengths ranging from 300-1200bp.

### Functional classification

All unigenes were aligned against public databases (NR, COG, and KEGG) using blast+ programs[17]. The results showed that 33,573 (68.23% of 49,207) unigenes were matched to one or more of the databases.

**Fig 1:**
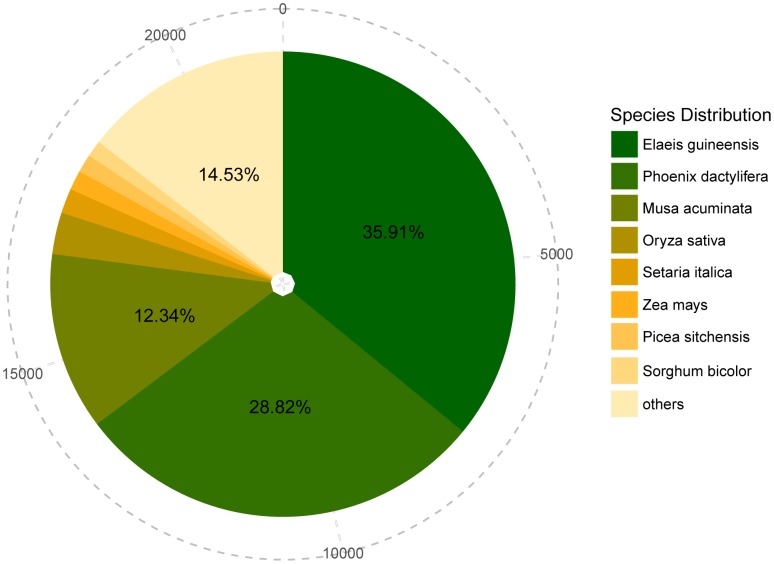
Species distribution of best blastx hits to NR database. Cumulative total numbers of unigenes annotated to NR are shown in the outermost circle with the dotted line. The size of the area isin proportion to the percentage of best blastx hits to the corresponding species.

According to all alignments, 21,508(47.58%) unigenes had homologous proteins in the NR database. Of these unigenes, 7,727(35.91%) were significantly similar to sequences of *Elaeis guineensis*, and 6,199(28.82%) and 2,655(12.34%) unigenes showed high similarity to sequences of *Phoenix dactylifera* and *Musa acuminata*, respectively (Fig 1).

**Fig 2:**
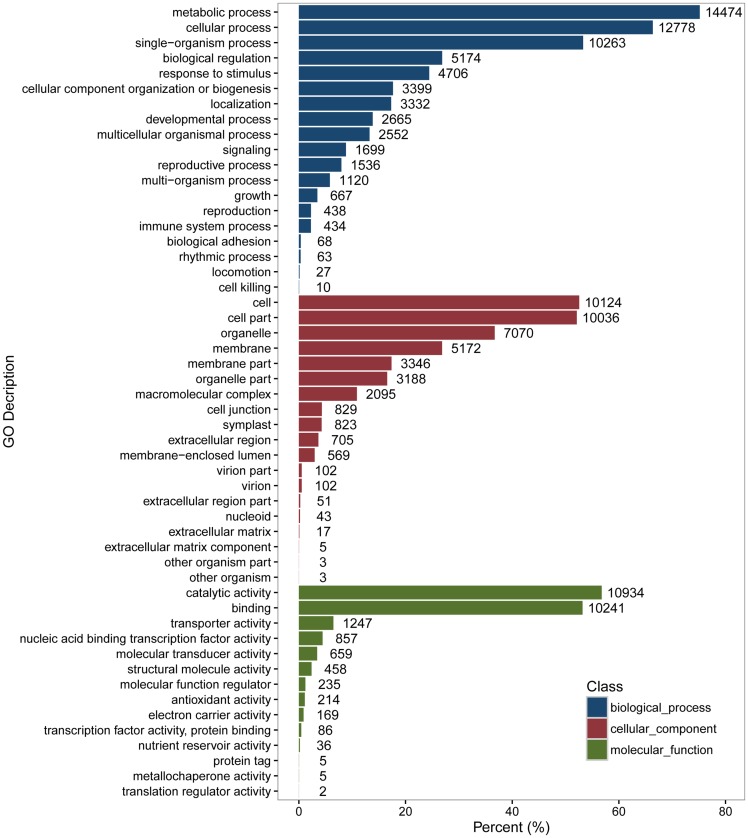
Gene ontology classification of all unigenes at level 2. The number of gene GO terms in each functional subcategory is presented as the percentage of GO terms for that subcategory out of the total GO terms.

Gene Ontology (http://geneontology.org/) provides controlled vocabularies of defined terms representing gene product properties, covering three domains: Cellular Component, Molecular Function, and Biological Process. Based on sequence homology, a total of 19,257(41.05%) unigenes were assigned to one or more GO terms (Fig 2). Among these terms, ‘metabolic process’, ‘cellular process’ and ‘single-organism process’ were the most representative terms in the biological process category. ‘Cell’, ‘cell part’ and ‘organelle’ were the terms that dominated in the cellular component category. ‘Catalytic activity’ and ‘binding’ were the most abundant terms in the molecular function category. Only a few unigenes (less than ten) were clustered into the terms of ‘translation regulator activity’, ‘metallochaperone activity’, ‘protein tag’, ‘other organism’, ‘other organism part’ and ‘cell killing’.

All unigenes were aligned against the Cluster of Orthologous Groups (COG) database for functional prediction and classification. In total, 11,871 (16.43% of 45,207) unigenes were assigned appropriate COG clusters, which could be classified into 25 functional categories (S3 Fig). Among them, the largest category was ‘General function prediction only’ (13.44%); followed by ‘signal transduction mechanisms’ (10.42%); ‘posttranslational modification, protein turnover, chaperones’ (7.76%); ‘cell wall/membrane/envelope biogenesis’ (7.61%); and ‘carbohydrate transport and metabolism’ (6.38%).

To identify the biological pathways activated in pineapple under cold stress, all unigenes were mapped to the KEGG database (http://genome.jp/kegg/). A total of 10,750 unigenes were significantly matched by homology in the KEGG database and were assigned to 139 KEGG pathways. A total of 3,711 (34.52%) unigenes were related to ‘carbohydrate metabolism’, while 2,746 (25.54%) and 2,269 (21.11%) unigenes participated in ‘translation’ and ‘lipid metabolism’. In addition, many unigenes were involved in pathways related to plant cold response, namely ‘plant-pathogen interaction’, ‘plant hormone signal transduction’ and ‘starch and sucrose metabolism’.

### Identification and validation of candidate cold-response genes (CORs) of pineapple

To find cold-response genes in pineapple, clean reads of the control and cold libraries were assigned to all unigenes using RSEM[18]. All resultant expected counts were then subjected to DEseq[19] to detect potential cold responsive genes (CORs). In total, 1,186 CORs were identified, with 898 unigenes up-regulated and 288 unigenes down-regulated. To confirm that the differentially expressed genes obtained in this study were credible, ten CORs were randomly selected and their expression patterns examined via RT-qPCR (S2 Table). The results showed that the expression profiles of all these CORs were similar to the ones obtained from the RNAseq results (S4 Fig), indicating that the method used to determine differentially expressed genes in this study was valid.

### Gene set functional enrichment analysis of CORs

The home made application ‘TBtools’ (http://cj-chen.github.io/tbtools/) was used to identify GO terms that were remarkably enriched in CORs. Enriched GO terms in up-regulated and down-regulated CORs were identified separately using the hyper-geometric test.

**Fig 3:**
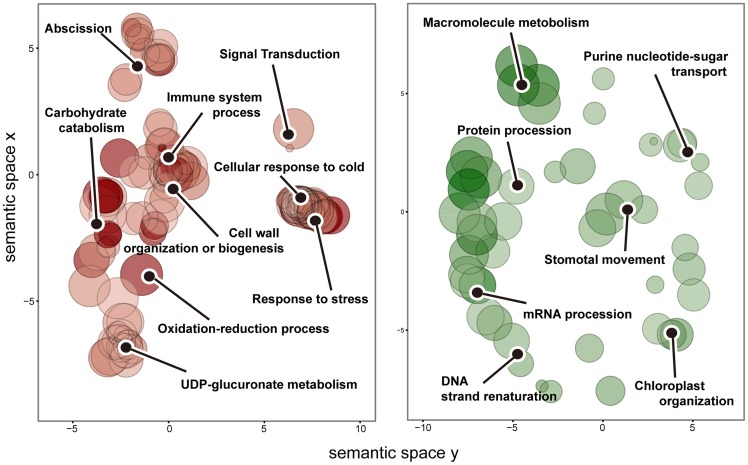
Significantly enriched GO terms in biological process category in a two-dimensional semantic space. Red-filled circles represent enriched GO terms of up-regulated CORs, and green-filled circles represent enriched GO terms of down-regulated CORs. Color intensity reflects the significance of enrichment test, with dark colors corresponding to lower P values and all P values lower than 0.05. Circle radiuses depict the sizes of the aggregated GO terms.

In the category of biological processes, the GO terms that were over-represented in up-regulated CORs and in down-regulated CORs were both obviously related to cold response (Fig 3). Among them, ‘cellular response to cold’, ‘response to stress’, ‘signal transduction’and‘abscission’were enriched in up-regulated CORs. ‘DNA strand renaturation’, ‘mRNA procession’ and ‘protein procession’, ‘stomatal movement’, ‘macromolecule metabolism’ and ‘purine nucleotide-sugar transport’ were over-represented in down-regulated CORs.

Regarding the molecular function category at gene ontology level 3, ‘transcription factor activity, sequence-specific DNA binding’ and ‘oxidoreductaseactivity’ were enriched in up-regulated CORs.‘Heterocyclic compound binding’ and ‘organic cyclic compound binding’ were enriched in down-regulated CORs.

Regarding thecellular component category, ‘extracellular region’, ‘ cell wall’, ‘external encapsulating structure’, ‘apoplast’, ‘cell periphery’ and ‘anchored component of membrane’ were enriched in up-regulated CORs, while only one GO term, ‘SWI/SNF superfamily-type complex’, was enriched in down-regulated CORs.

**Fig 4:**
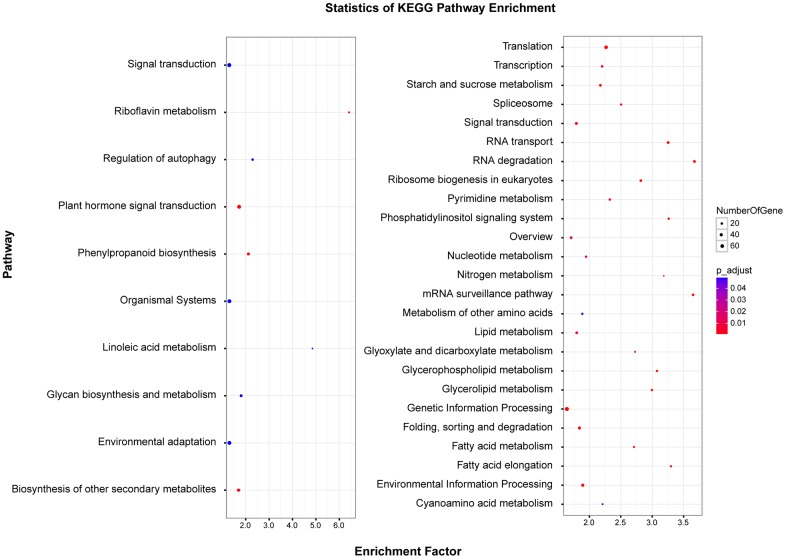
Enriched KEGG pathways for up-regulated CORs (left) and down-regulated CORs (right). Point size is correlated to the number of CORs involved in the corresponding pathways, with the color denoting the corrected p value.

Regarding KEGG pathways(Fig 4), ten pathways were observed to be significantly enriched for up-regulated CORs. ‘Riboflavin metabolism’ was the most significant one with the highest enrichment factor, followed by others that mightbe related to cold adaption, such as ‘environmental adaption’. Comparatively, a series of pathways were enriched in down-regulated CORs, including ‘translation’, ‘transcription’ and ‘starch and sucrose metabolism’.

### Prediction of protein association network in CORs

**Fig 5:**
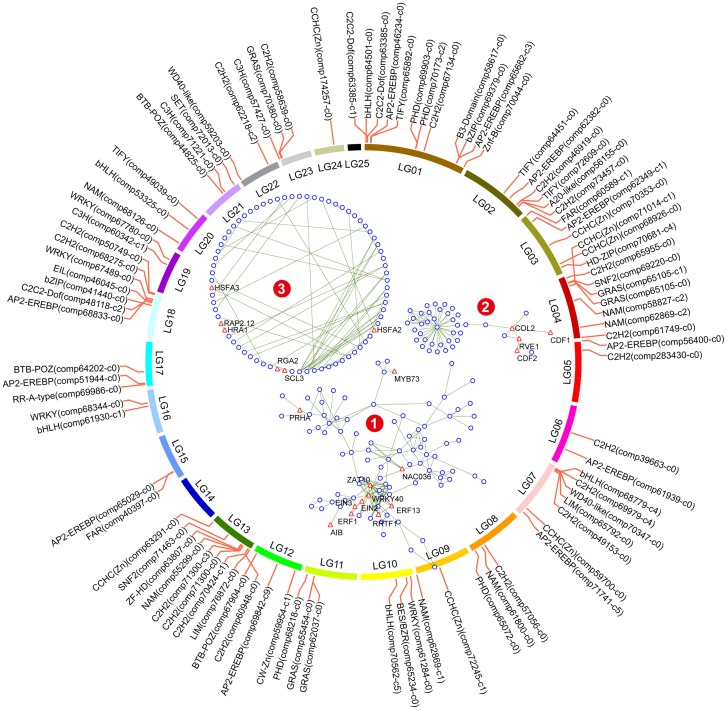
Distribution of cold-responsive transcription factors in pineapple (*Ananas comosus* var. F153) genome and putative protein association network of CORs. The outermost circle displays the 25 chromosomes of pineapple. The identified transcription factors in CORs are placed in their positions on the ideogram. The filtered protein association network is shown in the ideogram. Links between nodes indicate evidence of association between the two nodes in the String database with scores higher than 700. Transcription factors are shown as a triangle labeled with the gene name.

Protein-coding genes mainly function by interacting with other proteins or binding to the promoter regions of other genes. It was expected that CORs in this study would beassociatedwith other proteins and that transcription factors would playan important role. Thus, using *Arabidopsis thaliana* gene associations for reference, we inferred the protein association network of CORs (Fig 5). In total, 606 CORs were matched to 531 *Arabidopsis* proteins in the String database[20]. After filtering edges with scores below 700 and the resultant single nodes, a protein association network of 214 nodes with 286edges was obtained. Additionally, we predicted transcription factors in CORs using PlantTFcat[21]. In total, 116 differentially expressed transcription factors were identified and were clustered in 36 TF families, including MYB, AP2-EREBP and C2H2.

## Discussion

### Cell wall properties are critical to cold tolerance of pineapple

The plant cell wall is an extracellular matrix consisting of cellulose, hemicellulose and lignin. It plays essential roles in plant growth and in adaptive responses to adverse environmental conditions. Plant cell walls and cell tension have previously been reported to be associated with both cold acclimation in hardy plants [22,23] and cold tolerance in cold-sensitive plants. In *Arabidopsis*, a TCF1-depedent pathway was proposed[24]. TCF1 (Tolerant to Chilling and Freezing 1) modulates BCB (Blue Copper Binding protein) to adjust lignin accumulation and consequent cell wall remodeling, thereby increasing the freezing tolerance of the cell. In rice, specific expressed gene are functionally related to cellular cell wall organization in the cold-tolerant introgression line ‘K354’ compared to its cold-sensitive recurrent parent C418, which may contribute to increased cold tolerance[25]. Similarly, in this study, ‘cell wall organization and biogenesis’- and ‘UDP-glucuronate metabolism’-related genes were enriched among the up-regulated CORs. UDP-glucuronate is a precursor for hemicellulose, one of the main components of the cell wall[26]. The results indicated that a similar change in the cell wall of pineapple as in hardy plants occurs when subjected to cold stress[27].

### Stomatal closure related genes contribute to cold tolerance of pineapple

When a plant is exposed to cold stress, a number of biological processes are repressed. Rapid low temperature-induced stomatal closure occurs in cold-tolerant *Commelina communis* leaves but not in cold-sensitive tobacco leaves, depending on a mechanism that involves apoplastic calcium[28]. In *Arabidopsis*, stomatal closure confers abiotic stress tolerance[29]. In this study, ‘stomatal movement’ was enriched in down-regulated CORs, while ‘apoplast’ was enriched in up-regulated CORs, indicating that the same mechanism might contribute to cold-tolerance in pineapple and that these CORs, including comp59956_c0(CIPK14, Calcium sensor protein—interacting serine/threonine protein kinase), might be worth further research.

### ABA and ROS signal transduction play an important role in the cold response of pineapple

Many signal transduction cascades are involved in the low-temperature response process and are generally divided into two groups, ABA-dependent and ABA-independent[30,31]. ABA-dependent pathways are believed to be regulated by transcription factors belonging to the bZIP family, such as ABA-responsive element binding factors (AREBs)[32]. In this study, the GO term ‘abscission’ was significantly over-represented in up-regulated CORs. According to the KEGG pathway annotation, all CORs related to ABA signal transduction were up-regulated, including PYR/PYL homologous genes (comp62525_c0 and comp58539_c1) and PP2C homologous genes (comp49925_c0 and comp59129_c0). These result simplied that ABA-dependent pathways should play a predominant role in the cold tolerance of pineapple.

When a plant is threated by biotic or abiotic stress, ROS (reactive oxygen species) accumulate in cells. On the one hand, the excessive accumulation of ROS leads to cellular injury, which ultimately leads to the death of the plant due to damage to the photosynthesis system II reaction center and membrane lipids[30]. The plant defends itself through a sophisticated mechanism that scavenges reactive oxygen. In this study, ‘oxidation-reduction process’ was significantly over-represented in up-regulated CORs. On the other hand, ROS signaling serves as a key player in plant stress signaling[33–35]: it responds to Ca^2+^, activates MAPK pathways and oxidatively induces transcription factor expression[36,37]. Cheng *et al*. compared the chilling sensitivity between indica and japonica rice and proposed a hypothetical model of an ROS-mediated regulon (ROS-bZIP-as1/ocs) triggered by chilling stress[11,33]. Pineapple and rice are both cold-sensitive herbaceous monocots that occur in moderate climates. Considering the enrichment of genes with oxidation-related functions in CORs, a similar ROS-mediated model might also play an important role in pineapple cold response.

### Cold response of pineapple involves a complex protein association network

The CBF-dependent pathway is a key responder to low-temperature stress in plants[38]. In this study, CBF3 (comp71741_c5) was significantly up-regulated under cold stress. However, it was not included in the protein association network we predicted (Fig 5). Genes regulated by CBF proteins are important for cold tolerance, but they only account for a small percentage of the COR genes[39]. In *Arabidopsis*, approximately 1,200 genes were robustly regulated by low temperature, only 170 of which belonged to CBF regulons[40]. In addition, CBF-dependent pathways contribute less to cold tolerance in cold-sensitive plants than in hardy plants. Tomato has a functional CBF cold-response pathway, butits CBF regulons differ from the ones in freezing-tolerant *Arabidopsis*[41]. Rice has aproposed ROS-mediated model triggered by chilling stress that is independent of CBF[11,33]. Therefore, in pineapple, it seemed that while CBF3 might play a crucial role in cold tolerance, other mechanisms might have make greater contributions and deserve deeper concern.

Based on the associations between the CORs (Fig 5), the putative network could be roughly divided into three sub-clusters. The CORs in sub-cluster 1 and sub-cluster 2 were well clustered separately, while the remaining CORs, each of which possessed at least one neighbor, formed sub-cluster 3. Transcription factors activate and repress the transcription of genes, playing central roles in gene regulatory networks. As reflected by the well-known functions of transcription factors in the protein association network (Table 1), sub-cluster 1 was associated with cold stress and functioned in the signal transduction of several plant hormones, including ABA, JA, ET and SA. Sub-cluster 2 was related to mechanisms such as cold acclimation, while sub-cluster 3 responded to ROS and low oxygen stress and participated in GA signaling.

**Table 1:** Functions of transcription factors in protein association network.

Cluster_ID	Unigene_ID	Gene_Name	Functional_Description
Sub_cluster_1	comp70630_c0	*AIB*	negatively regulates JA signaling [42] and positively regulate ABA signaling [43]
	comp46045_c0	*EIN2*	modulates stress response through ABA response pathway [44]
	comp69602_c0	*EIN3*	modulates JA and ET signaling antagonism in *Arabidopsis thaliana*[45]
	comp126555_c0	*ERF1*	enhances cold tolerance in tobacco [46]
	comp62349_c1	*ERF1*	
	comp64343_c5	*ERF13*	responds to ethylene signaling*
	comp61939_c0	*MYB73*	early cold-induced under cold stress [40]
	comp62382_c1	*NAC036*	positively regulates SA-mediated responses[44]
	comp70562_c5	*PRHA*	positively influenced by the phytohormone auxin*
	comp67780_c0	*RRTF1*	maintains the redox responsiveness of the network [47]
	comp61800_c0	*RRTF1*	
	comp55915_c0	*WRKY40*	regulates ABA signaling regulators*
	comp69979_c4	*ZAT10*	early cold-induced under cold stress [40]
Sub_cluster_2	comp63385_c1	*CDF1*	controls CO expression downstream of photoreceptors[48]
	comp48118_c2	*CDF2*	controls plant development [49]
	comp63385_c0	*CDF2*	
	comp65390_c1	*COL2*	up-regulated in cold acclimation of *Arabidopsis* and down-regulated in deacclimation[50]
	comp69692_c0	*RVE1*	negatively regulates cold acclimation in *Arabidopsis*[51]
Sub_cluster_3	comp67661_c1	*HSFA2*	increases tolerance to abiotic stress [52,53]
	comp51907_c0	*HSFA3*	increases heat and oxidative tolerance [54]
	comp62904_c0	*HSFA3*	
	comp60887_c1	*HRA1*	induced by low oxygen*
	comp65029_c1	*RAP2*.*12*	dynamically regulated by oxygen concentration*
	comp55454_c0	*RGA2*	negatively regulates GA responses[55]
	comp65105_c0	*SCL3*	positively regulates GA signaling[56]
	comp65105_c1	*SCL3*	

* Functionaldescriptions with no reference were obtained from theTAIR gene database (https://www.Arabidopsis.org/).

Pineapple, unlike hardy plants, does not possess a functional cold acclimation mechanism, whereas genes involved in this mechanism might also contribute to its cold tolerance. The ABA-dependent pathway has been shown to be an important part of cold response in plants. CORs and their associations in sub-cluster 1 will be helpful in unraveling the related mechanisms and determining how to increase the cold tolerance of pineapple. Moreover, as further studies are conducted, the cold response network will become increasingly complex[39]. The associations between CORs in sub-cluster 3 will be elaborated, and this cluster will serve as a reference for studies that focus on ROS and GA signaling in the cold response of pineapple.

Therefore, the gene network involved in the cold response of pineapple is complex and requires further research. The putative clusters in this study would be helpful in dissecting it.

## Conclusion

The pineapple transcriptome under cold stress was analyzed using RNAseq. A total of 1,186 CORs were identified, with 898 unigenes up-regulated and 288 unigenes down-regulated. The credibility has been validated by RT-qPCR. As reflected by functional analysis, genes related to cell wall properties, stomatal closure and ABA and ROS signal transduction contribute to the cold tolerance of pineapple. While CBFs might also play important roles, the more complex network deserves more attention. The CORs identified in this study will serve as entry points to dissect this cold responsive network in pineapple. In short, these results will be helpful for understanding the cold-response mechanism of pineapple and contribute to pineapple breading for cold tolerance.

## Methods and Materials

### Plant materials and cold treatment

All pineapple plantlets (*Ananas comosus* cv.‘Shenwan’) were obtained from tissue culture generation of the same sucker whose maternal plant survived a strong cold wave that shocked Guangzhou in 2008 and was able to grow normally after cold exposure to 4°C for up to 7 days. The plantlets were maintained in a climate chamber where the temperature was maintained at 26°C. A total of60plantlets from three batches of tissue culture generation (each with 20 plantlets) at similar growth stages were selected before and after cold treatment(4°C 24h). Before cold treatment, three groups of leaves were collected from three batches of tissue culture generation (each with 10 plantlets), forming biological replicates of sample group I (Control_1, Control_2, Control_3a). After cold treatment, three more groups were collected from the three corresponding batches of tissue culture generation, forming the biological replicates of sample group II (Cold_1, Cold _2, Cold_3a). All samples were immersed in liquid nitrogen and stored at -80°C for RNA extraction. In each group, RNA was separated from one batch (Control_3a, Cold_3a) after RNA extraction, forming two technological replicates (Control_3b, Cold_3b). In addition, the same preparation procedure was repeated for the RT-qPCR experiment.

### RNA extraction, cDNA library construction, and sequencing

The total RNA from each sample was extracted using the TRIzol reagent (Invitrogen). An Agilent 2100 Bioanalyzer was used to test the integrity of the RNAand revealed that all RNA samples were integrated (integrity number value > 8.0). Equal amounts of all RNA samples were pooled, forming the library pool. All libraries were prepared according to the manufacturer’s instructions of the Truseq2 RNA sample prep Kit (Illumina, Inc. San Diego, CA, USA). First, mRNA was enriched using magnetic beads containing poly-T molecules. Subsequently, the enriched mRNA was broken into short fragments and then reverse transcribed into cDNA using the PrimeScript 1st Strand cDNA Synthesis Kit (Takara). Finally, these cDNA fragments underwent end repair and were ligated using Illumina adapters. Three libraries were then sequenced using the IlluminaHiSeq^™^2000. The raw data were deposited in the NCBI Sequence Read Archiveunder accession numbersSRP079813.

### Quality control, *de novo* construction of transcripts

The raw data (raw reads) in fastq format were first processed by Trimmomatic[57]. In this step, high-quality reads were obtained by removing reads containing adapters, reads containing poly-N and low-quality reads. At the same time, the Q20, Q30 and GC content were calculated. Then, bwa[58] was employed to align all high-quality reads against the silva database[59]. Next, rRNA reads were removed, and clean data were obtained. The downstream analyses were based on the clean high-quality data. Reads from all libraries were *de novo* assembled using Trinity[15] and CD-hit[16]into a gene set that served as the reference for subsequent analysis.

### Functional annotation and classification

All genes were aligned against public data bases (Nr, COG, KEGG) to obtain their putative functions, leveraging blast+ with evalue< = 1e-5 and query coverage > = 0.33. Based on Nr annotation, the species list of all best hits (highest bit scores in all alignment) were extracted for further analysis, and the GI list was transformed into Gene Ontology numbers using the home made application TBtools(http://cj-chen.github.io/TBtools/).

### Differential expression analysis and functional enrichment analysis

The differential expression analysis of the two conditions was performed using DESeq[19]. The P values were adjusted using the Benjamini & Hochberg method. The corrected P-value of 0.05 and abs(log2(Fold change)) of 1 were set as the threshold for significantly differential expression. GO and KEGG pathway enrichment analysis were performed by TBtools using the hyper-geometric test. Revigo[60] was applied to visualize the GO enrichment results.

### Protein association network prediction and transcription factor identification

The protein sequences and protein association information of *Arabidopsis thaliana* were downloaded from String databases[20], which were used as reference. The protein association network of the CORs was then inferred after analysis of the blastx results and visualized using Cytoscape[61]. All transcription factors were predicted by PlantTFcat[21], and their positions in the genome were visualized using Circos[62].

## Supporting Information

S1 Fig Sample clustering based on log2-transform of RPKM values. Samples were clustered based on log2(RPKM) value using the R functions ‘cor’ with the ‘spearman’ method and ‘hclust’ with the ‘average’ method.
(TIF)

S2 Fig Length distribution of unigenes and predicted CDS. The x-axis denotes the length range of all groups. The y-axis denotes the number of unigenes and CDSs in each group. CDSs with lengthsunder 300 bp were filtered in the prediction procedure.
(TIF)

S3 Fig COG classification of unigenes. Unigenes were clustered into 25 COG categories. The y-axis denotes the number of unigenes in each group. The x-axis denotes the functional description of each group. Details are shown in the right part of the graph.
(TIF)

S4 Fig Expression levels of ten unigenes in RNAseq and RT-qPCR experiment. Line plots displaying the RPKM values of ten unigenes in RNAseq experiments. Bar plots with errorbars display the relative expression values in the RT-qPCR experiments.
(TIF)

S1 Table Detailed information on cDNA libraries. (XLSX)

S2 Table Primers used in RT-qPCR experiments. (XLSX)

S3 Table Transcription factors in putative protein association network. (XLSX)

S4 Table Gene annotation of all CORs. (XLSX)
