# Supplementary figures and images for: Transcriptome Profiling of the Pineapple under Low Temperature to Facilitate Its Breeding for Cold Tolerance
Source: PLoS One. 2016 Sep 22;11(9):e0163315. doi: 10.1371/journal.pone.0163315 (PMC5033252; doi:10.1371/journal.pone.0163315)

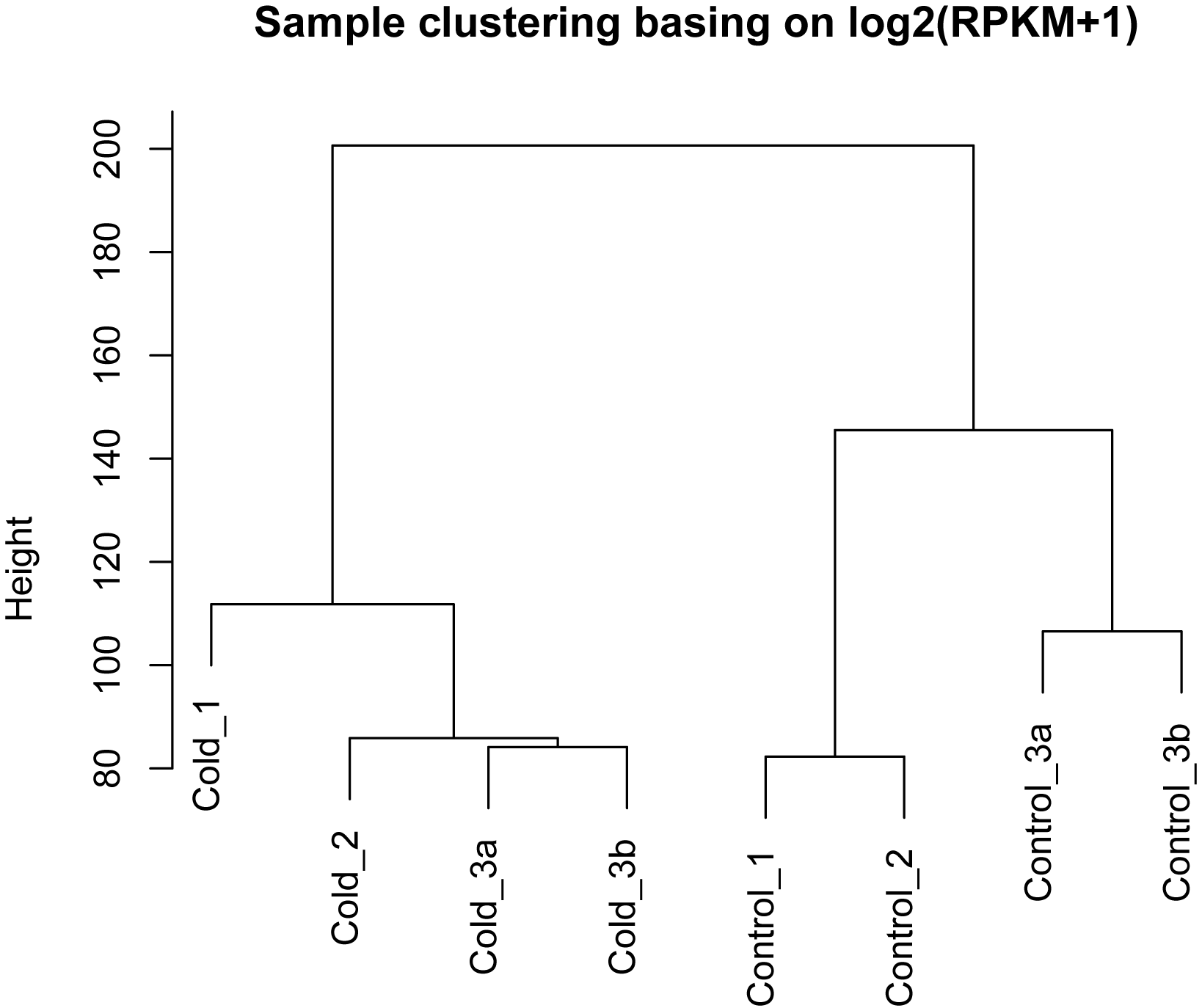

Supplement: S1 Fig — Samples were clustered based on log2(RPKM) value using the R functions ‘cor’ with the ‘spearman’ method and ‘hclust’ with the ‘average’ method. (TIF) [file pone.0163315.s001.tif]

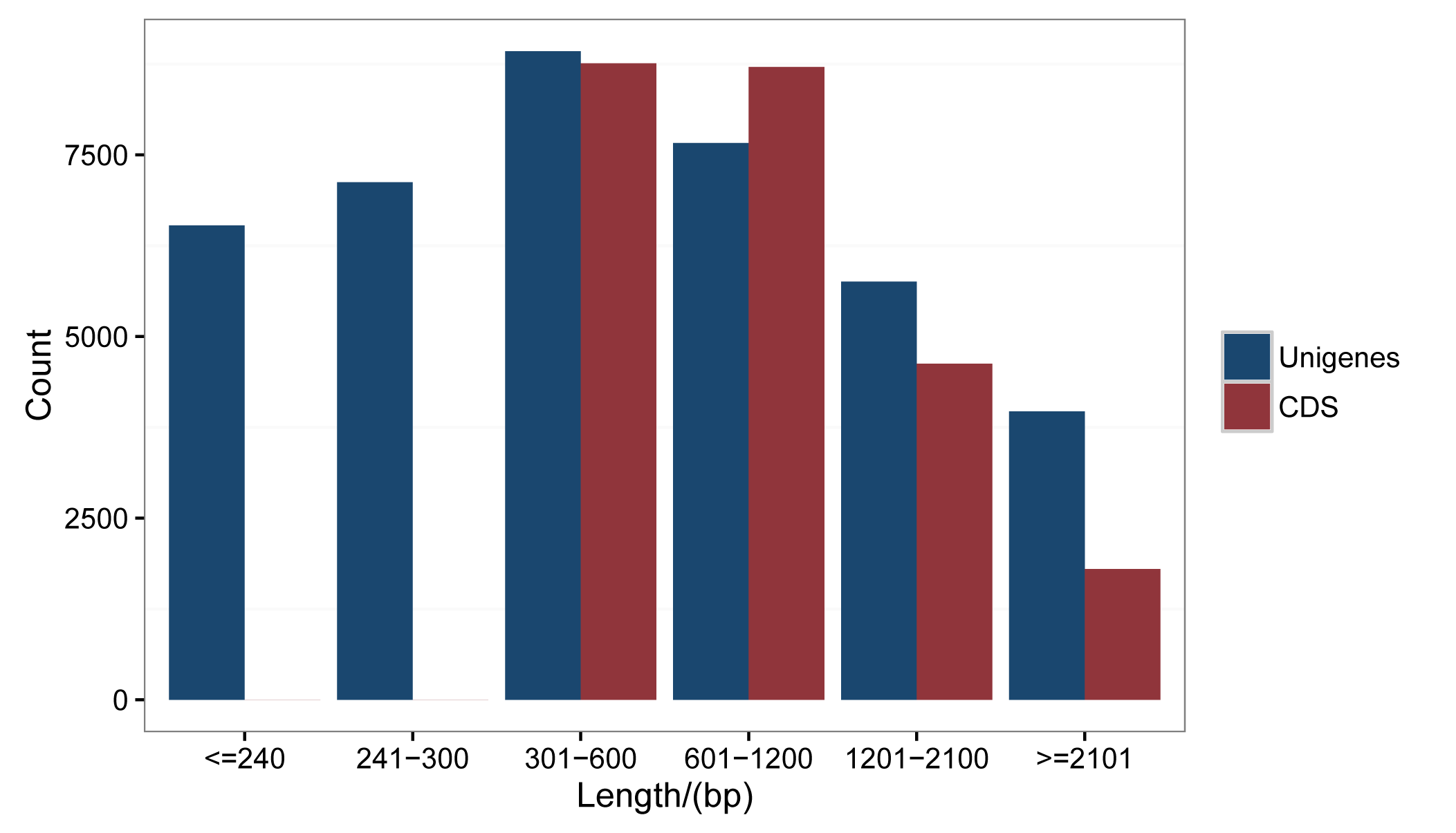

Supplement: S2 Fig — The x-axis denotes the length range of all groups. The y-axis denotes the number of unigenes and CDSs in each group. CDSs with lengthsunder 300 bp were filtered in the prediction procedure. (TIF) [file pone.0163315.s002.tif]

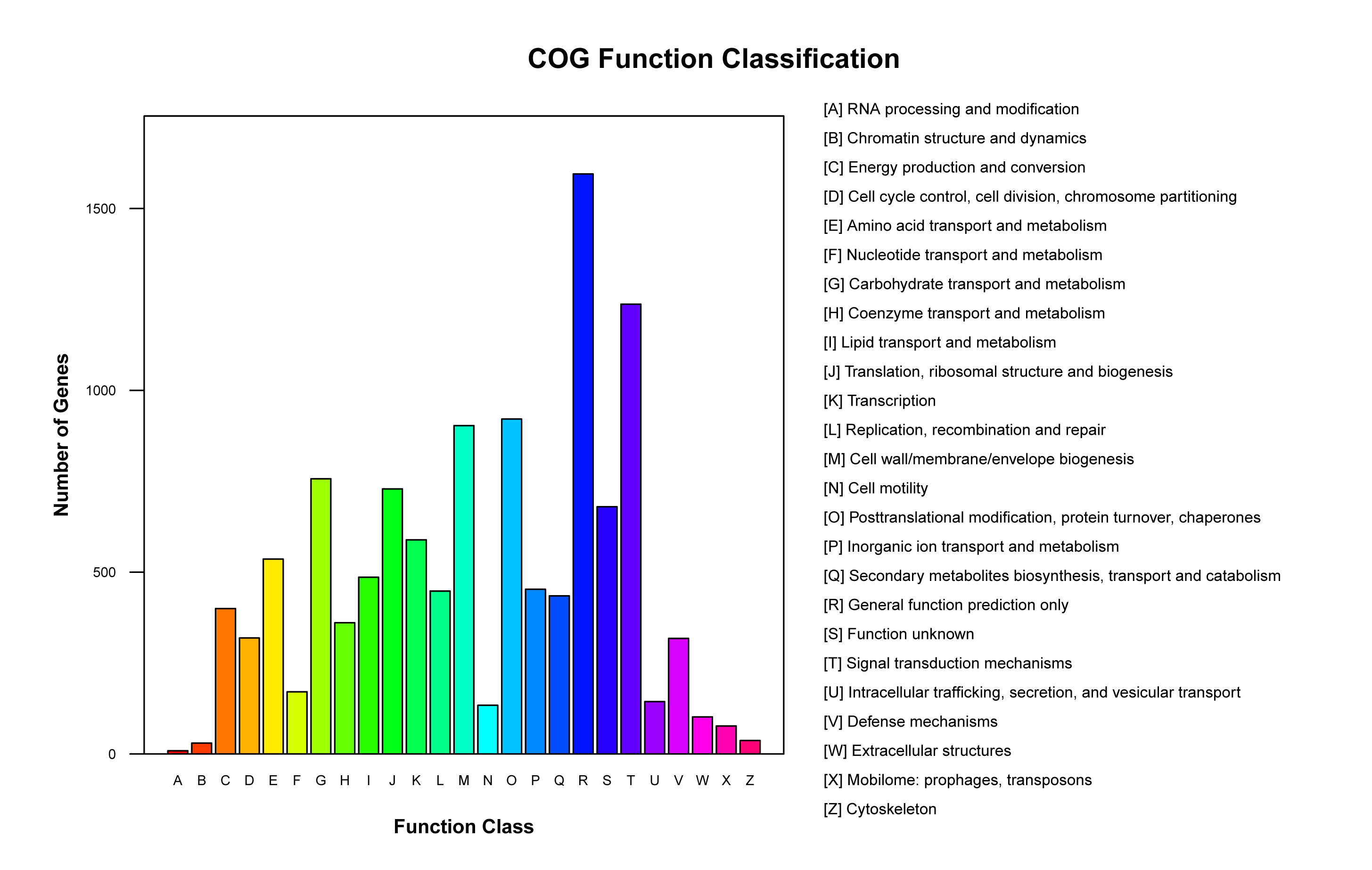

Supplement: S3 Fig — Unigenes were clustered into 25 COG categories. The y-axis denotes the number of unigenes in each group. The x-axis denotes the functional description of each group. Details are shown in the right part of the graph. (TIF) [file pone.0163315.s003.tif]

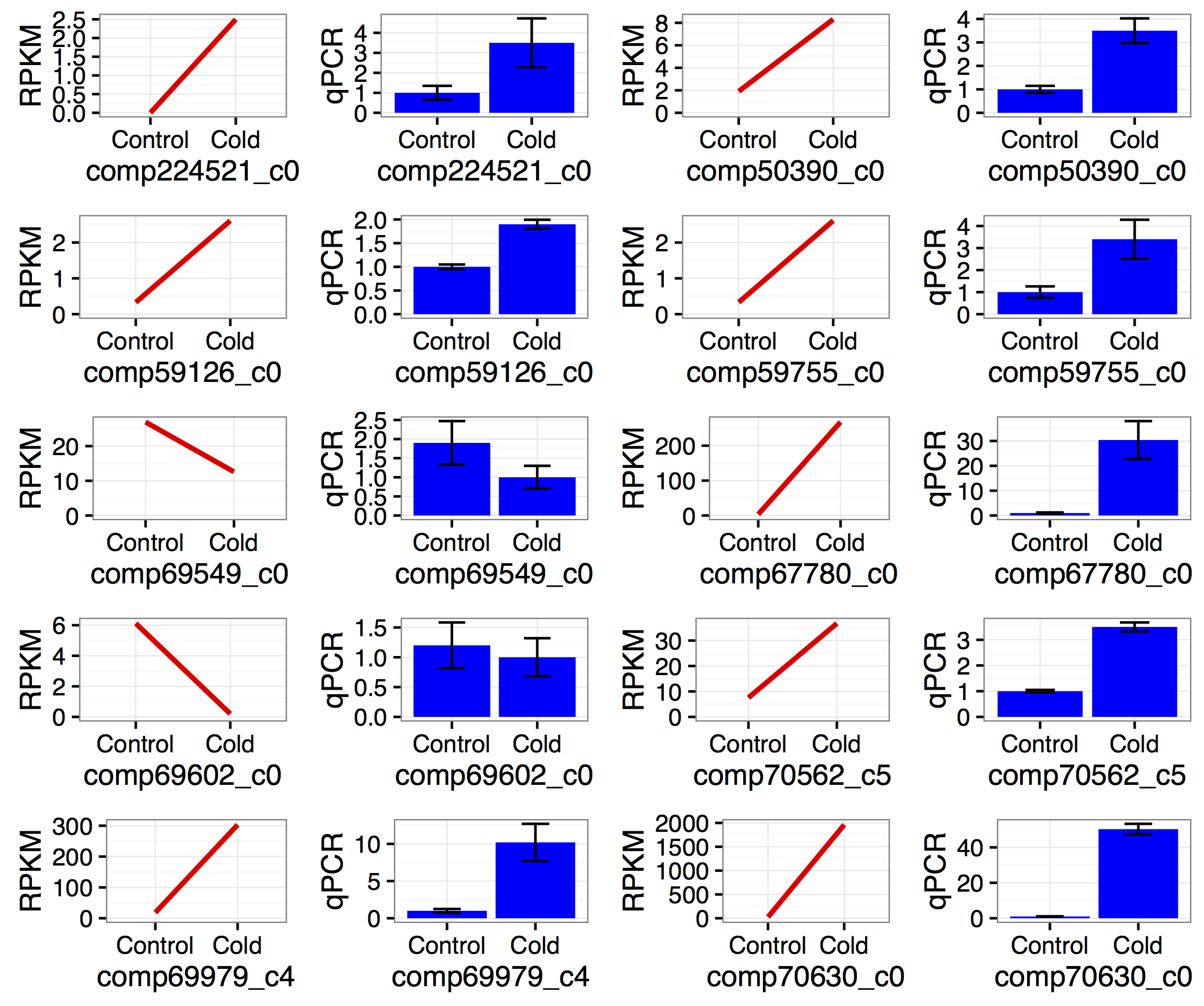

Supplement: S4 Fig — Line plots displaying the RPKM values of ten unigenes in RNAseq experiments. Bar plots with errorbars display the relative expression values in the RT-qPCR experiments. (TIF) [file pone.0163315.s004.tif]
